# Free Wanderer Powder regulates AMPA receptor homeostasis in chronic restraint stress-induced rat model of depression with liver-depression and spleen-deficiency syndrome

**DOI:** 10.18632/aging.103912

**Published:** 2020-10-14

**Authors:** Shengyan Xi, Guangxin Yue, Yueyun Liu, Yanan Wang, Yingkun Qiu, Zhigeng Li, Pei Ma, Tiegang Liu, Youming Jiang, Yuan Liang, Qun Liu, Jing Shi, Jiaxu Chen, Lifeng Yue

**Affiliations:** 1 Department of Traditional Chinese Medicine, School of Medicine, Xiamen University, Xiamen 361102, China; 2 China Academy of Chinese Medical Sciences, Beijing 100700, China; 3 School of Traditional Chinese Medicine, Beijing University of Chinese Medicine, Beijing 100029, China; 4 School of Pharmaceutical Sciences, Xiamen University, Xiamen 361102, China; 5 Institute of Medicinal Plant Development, Chinese Academy of Medical Sciences and Peking Union Medical College, Beijing 100193, China; 6 The 3rd Neurology Department, Dongzhimen Hospital, Beijing University of Chinese Medicine, Beijing 100700, China

**Keywords:** depression, free wanderer powder, AMPA receptor, homeostasis, chronic restraint stress

## Abstract

Free Wanderer Powder (FWP) is a classic formula for depression with digestive dysfunctions, i.e., liver-depression and spleen-deficiency syndrome (LDSDS) in Chinese Medicine. But its protective mechanism has not been fully clarified. Here a chronic restraint stress (CRS) induced rat model showed depression with LDSDS in food intake, metabolism, and behaviour tests. Then 75 rats were randomly divided, and received CRS and different treatment with behaviour tests. Expressions of c-Fos and AMPA-type glutamate receptor subunits GluR1-3 in hippocampus CA1, CA3, DG and amygdala BLA were detected by immunohistochemistry, western blot and RT-PCR, respectively. In CRS rats, FWP alleviated depressive behaviour and c-Fos expression. FWP suppressed the increasement of GluR1 in CA1 and DG, p-GluR1 in CA1, and p-GluR2 and GluR3 in BLA. FWP also blocked the decrease of GluR1 and Glur2/3 in CA3, p-GluR1 in CA3, and p-GluR2 in CA1 and CA3. Furthermore, constituents of FWP and their potential targets were explored using UHPLC-MS and systematic bioinformatics analysis. There were 23 constituents identified in FWP, 9 of which regulated glutamatergic synapse. Together, these results suggest that FWP contains effective constituents and alleviates depression with LDSDS by regulating AMPA-type glutamate receptor homeostasis in amygdala and hippocampus.

## INTRODUCTION

Depression is a global life-threatening psychiatric disorder. Central to it is persistent depressed mood and/or loss of pleasure in most activities [1]. The somatic manifestations are commonly interpreted as "secondary" or "non-specific" dysfunctions [2]. In fact, two-thirds of primary care patients with depression present with somatic symptoms, including pain (joint, limb, back, and etc.), fatigue, psychomotor activity changes, appetite changes, and gastrointestinal problems [3]. Therefore, treatment focused on core emotional symptoms only could result in an incomplete remission and a poor treatment prognosis for the patient. Considering depression symptoms improve when physical symptoms alleviate [3], treatment modulating emotional and somatic symptoms at the same time is a good choice for depression.

The Traditional Chinese Medicine (TCM) theory has been used for primary care in East Asia for centuries [4]. Its accumulated experience supports new hypotheses in the West that treat depression as psychobiological symptom rather than psychological symptom [2]. In TCM, liver-depression and spleen-deficiency syndrome (LDSDS) is a main depression subtype caused by stagnation of liver qi and subsequent dysfunction of spleen [5, 6]. Five visceral organs (liver, heart, spleen, lungs and kidneys) in TCM describe the individual anatomico-physiological-psychological systems that constitute the total human being, rather than a strictly anatomical sense of organ [7]. The liver controls the spiritual soul and spleen controls digestive functions. Therefore, physical symptoms in LDSDS are presented as poor appetite and digestion, pain in rib-side and abdomen, uncomfortable loose bowels, or alleviation of abdominal pain after defecation. Psychological symptoms in LDSDS are presented as hypochondriac symptoms, depressed mood, and frequent sighing. Thus, experienced and effective formula for LDSDS in TCM, is a potential treatment and worthy of clarifying mechanisms. One of the most convinced formula is Free Wanderer Powder (FWP), which is used for depression with LDSDS for centuries.

Free Wanderer Powder (FWP) is recorded in the book of “Tài Píng Huì Mín Hé Jì Jú Fāng” (960-1127 AD), which consists of: chief herb-Radix Bupleuri, deputy herbs-Radix Angelicae Sinensis and Radix Paeoniae Alba, and assistant herbs-Rhizoma Atractylodis Macrocephalae, Poria, Rhizoma Zingiberis Recens, Herba Menthae and Radix et Rhizoma Glycyrrhizae Praeparata cum Melle [8, 9]. Previous studies have shown that FWP regulates brain regions such as the hippocampus, amygdala, locus coeruleus, and hypothalamus [10]. FWP has effects on tryptophan metabolism, BDNF signaling, kynurenine metabolism, apelin-APJ system and HPA axis in depression-like rats [11]. FWP is also used for digestive system and endocrine diseases [12], which is partially related to its regulation on 5-hydroxytryptophan [13]. Recently, FWP shows a hepatoprotective effects via regulating glutamine and glutamate metabolism to change levels of AST, ALT, SOD, MDA, CD68, and TNF-α in the liver [11]. However, the underlying mechanisms of FWP on LDSDS have not been systematically characterized.

Hippocampus and amygdala, the main structures within the limbic system, is a critical region in stress-related depression via regulating emotion, memory, motivation, autonomic, and endocrine function [14]. The enhanced glutamate levels induced by stress, inhibit neurons in hippocampus, excite neurons in amygdala BLA region, and are projected to the hypothalamus and the nucleus accumbens [15, 16]. Ionotropic glutamate receptors of the amino-3-hydroxy-5-methylisoxazole-4-propionic acid (AMPA) subtype are the main mediators for synaptogenesis, formation of neural pathways and synaptic plasticity in hippocampus and BLA [17]. Thus AMPA receptors (AMPARs) in hippocampus and BLA may be the major lesion for depression with LDSDS. Besides, heteromeric AMPA, consisting of four subunits GluR1-4, have altered expression, phosphorylation, and subcellular localization in several limbic regions in stressed animals [18]. However, a causal relationship between changes in AMPAR-mediated synaptic responses in different limbic regions and depression alleviation by FWP treatment has not been adequate.

Therefore, to fully investigate the effect of FWP on depression with LDSDS via regulating AMPAR receptor homeostasis, we first identified constituents of FWP by UHPLC-MS, then analyzed potential effective constituents/herbs on glutamatergic synapse via systematic bioinformatics analysis. Additionally, we evaluated the chronic restraint stress (CRS)-induced rat as a model of depression with LDSDS, and the effects of FWP on expressions of different AMPAR subunits, as well as c-Fos in hippocampus and BLA. We hope to demonstrate the effect of AMPAR homeostasis on depression with LDSDS, and find the potential anti-depressive mechanism of FWP.

## RESULTS

### Identification of compounds in FWP

**Figure 1:**
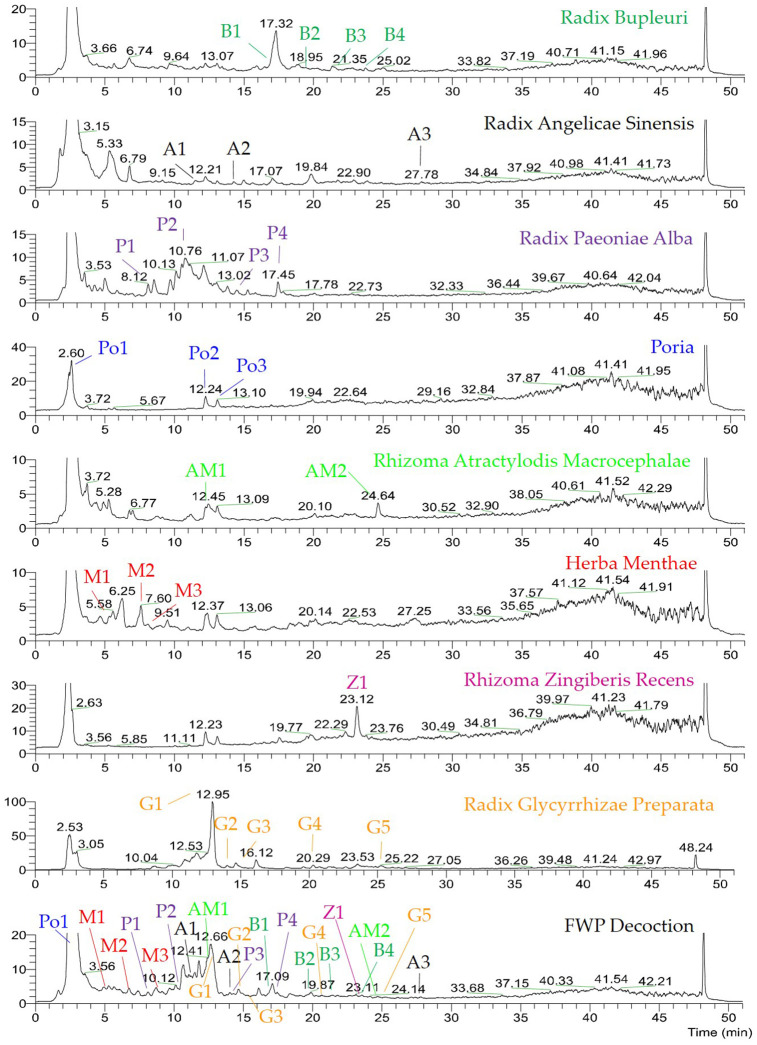
**UHPLC-MS chemical fingerprint of FWP.** D-Fructose (Po1), Lithospermic acid (M1), Rosmarinic acid (M2), Oxypaeoniflorin (P1), 3,4-Dihydroxybenzaldehyde (M3), Albiflorin (P2), Anisic acid (A1), 7-Hydroxycoumarin (AM1), Glycyrrhizic acid (G1), Angelol A (A2), Lactiflorin (P3), Ononin (G2), 4',7-Dihydroxyflavone (G3), Saikosaponin C (B1), Benzoylpaeoniflorin (P4), Saikosaponin F (B2), Formononetin (G4), Saikosaponin A (B3), 6-Gingerol (Z1), Saikosaponin E (B4), Licoflavone A (G5), Atractylenolide III (AM2) and Ligustilide (A3).

**Figure 2:**
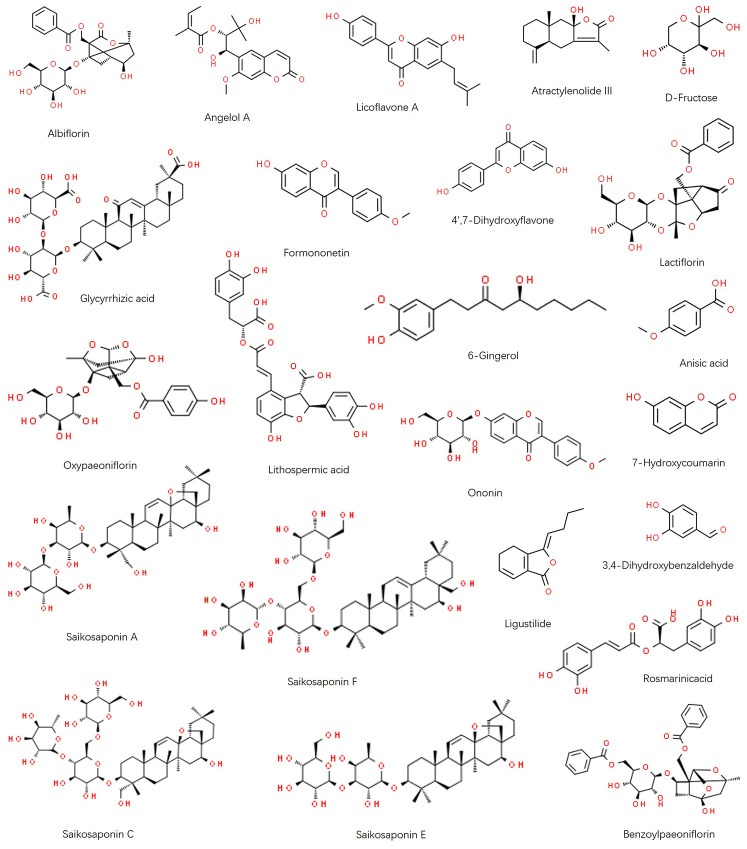
**Chemical structure of identified components in FWP: Albiflorin, Angelol A, Licoflavone A, Atractylenolide III, D-Fructose, Glycyrrhizic acid, Formononetin, 4',7-Dihydroxyflavone, Lactiflorin, Oxypaeoniflorin, Lithospermic acid, 6-Gingerol, Anisic acid, Ononin, 7-Hydroxycoumarin, Saikosaponin A, Saikosaponin F, Ligustilide, 3,4-Dihydroxybenzaldehyde, Rosmarinic acid, Saikosaponin C, Saikosaponin E, and Benzoylpaeoniflorin.**

As shown in Figure 1, the chromatographic fingerprints were constructed firstly for FWP and each herb. Then 23 peaks were identified by comparing their UV and MS spectra with published data or standard compounds (Table 1, Figure 2): D-Fructose (Po1), Lithospermic acid (M1), Rosmarinic acid (M2), Oxypaeoniflorin (P1), 3,4-Dihydroxybenzaldehyde (M3), Albiflorin (P2), Anisic acid (A1), 7-Hydroxycoumarin (AM1), Glycyrrhizic acid (G1), Angelol A (A2), Lactiflorin (P3), Ononin (G2), 4',7-Dihydroxyflavone (G3), Saikosaponin C (B1), Benzoylpaeoniflorin (P4), Saikosaponin F (B2), Formononetin (G4), Saikosaponin A (B3), 6-Gingerol (Z1), Saikosaponin E (B4), Licoflavone A (G5), Atractylenolide III (AM2) and Ligustilide (A3).

**Table 1:** Components in FWP identified by UHPLC-MS.

**NO**	**Name**	**ESI ion peaks in Herb**			**ESI ion peaks in Formula**		
		**tR (min)**	**ESI^-^**	ESI^+^ *	**tR (min)**	**ESI^-^**	ESI^+^ *
B1	Saikosaponin C	17.05	941.5145	965.5095	17.05	941.5144	965.5097
B2	Saikosaponin F	19.45	927.5307	951.5263	19.46	927.5306	951.5255
B3	Saikosaponin A	21.33	779.4616	803.4529	21.36	779.4618	803.4531
B4	Saikosaponin E	23.60	763.4650	787.4602	23.63	763.4644	787.4594
A1	Anisic acid	11.84	151.0389	175.1194	11.86	151.0389	175.1189
A2	Angelol A	14.23	375.1456	399.1424	14.35	-	399.1412
A3	Ligustilide	27.78	189.0917	191.1067	27.77	189.0917	191.1067
P1	Oxypaeoniflorin	8.10	495.1503	519.1487	8.12	495.1505	519.1491
P2	Albiflorin	10.78	479.1560	481.1715,503.1536	10.60	479.1559	503.1509
P3	Lactiflorin	14.47	461.1452	485.1435	14.45	461.1457	485.1412
P4	Benzoylpaeoniflorin	17.45	583.1833	607.1782	17.46	583.1833	607.1790
Po1	D-Fructose	2.58	179.0554	203.0527	2.56	179.0553	-
Po2	Unknown 1	12.30	564.4125	566.4285, 588.4077	12.24	564.4125	566.4289, 588.4082
Po3	Unknown 2	13.10	677.4990	679.5106, 701.4916	13.09	677.4989	679.5101, 701.4911
AM1	7-Hydroxycoumarin	12.47	161.0233	-	12.64	161.0234	-
AM2	Atractylenolide III	24.66	247.1335	-	24.65	247.1335	-
M1	Lithospermic acid	5.27	537.1057	-	5.14	537.1058	-
M2	Rosmarinic acid	7.62	359.0782	-	7.45	359.0781	-
M3	3,4-Dihydroxybenzaldehyde	8.74	137.0235	139.0180	8.68	137.0231	-
Z1	6-Gingerol	23.12	293.1760	317.1721	23.15	293.1762	317.1723
G1	Glycyrrhizic acid	12.91	821.3967	-	12.64	821.3969	-
G2	Ononin	14.87	-	431.1337, 453.1148	14.85	-	431.1337, 453.1149
G3	4',7-Dihydroxyflavone	15.36	253.0509	255.0656, 277.0472	15.33	253.0509	255.0655
G4	Formononetin	20.78	267.0664	269.0812, 291.0624	20.77	267.0664	269.0812, 291.0625
G5	Licoflavone A	24.46	353.1035	355.1178	24.47	353.1036	-

* [M+H]^+^ or [M+Na]^+^.

### The systematic bioinformatics analysis for antidepressive mechanism of FWP

**Figure 3:**
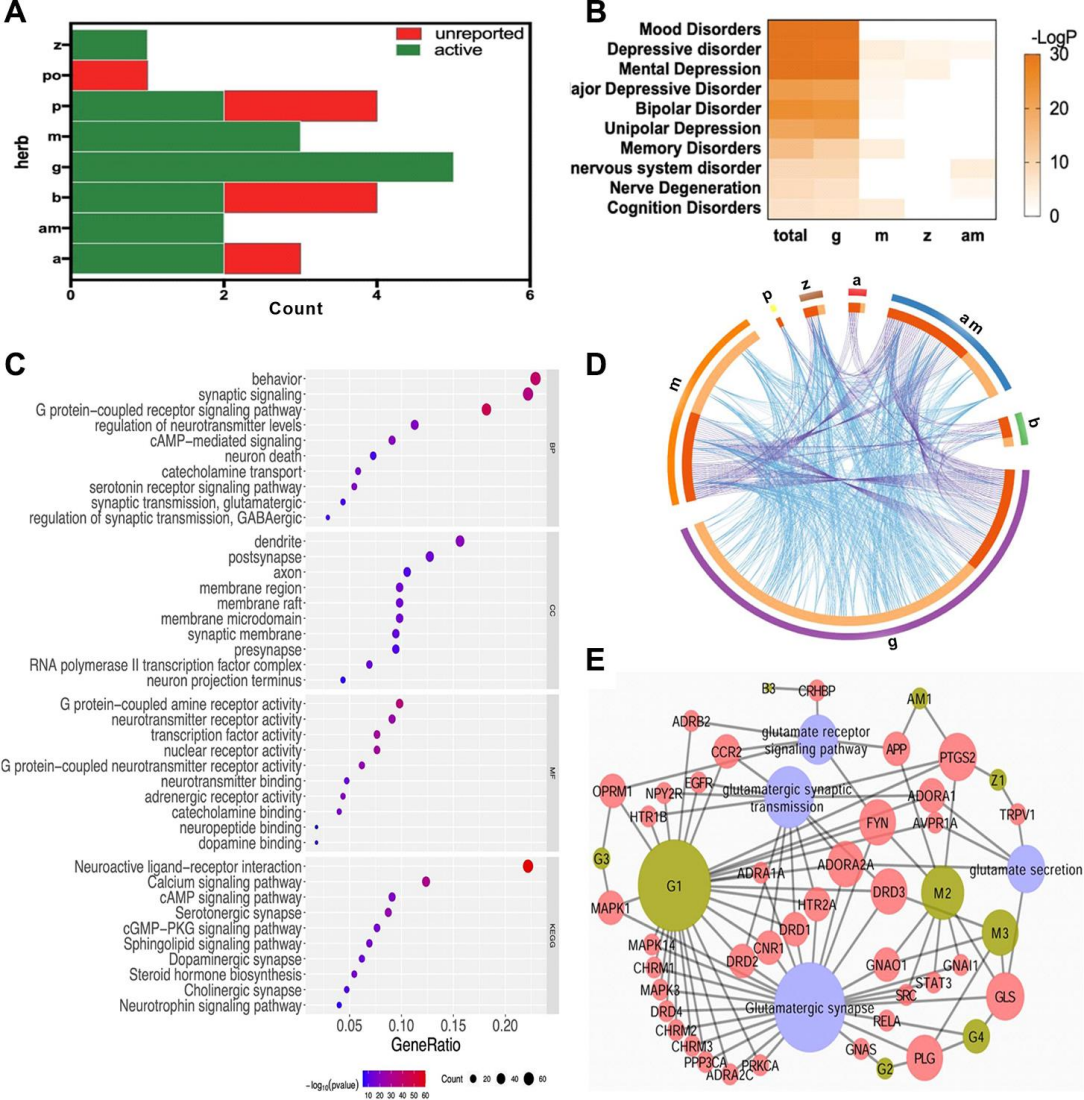
**The systematic bioinformatics analysis for antidepressive mechanism of FWP.** (**A**) compounds of FWP included (green) or unreported (red) in NPASS and PubChem database. (**B**) Enrichment analysis for herb-disease correlation (total: FWP). (**C**) Functional enrichment analysis on GO and KEGG for targets of FWP. (**D**) Target overlaps (purple line) and functional interaction (blue line) among targets for each herb in FWP. (**E**) Compound-Target-Pathway Network for compounds of FWP.

Totally, 17 compounds were collected, including 2 in Radix Angelicae Sinensis (a), 2 in Rhizoma Atractylodis Macrocephalae (am), 2 in Radix Bupleuri (b), 5 in Radix et Rhizoma Glycyrrhizae Praeparata cum Melle (g), 3 in Herba Menthae (m), 2 in Radix Paeoniae Alba (p), and 1 in Rhizoma Zingiberis Recens (z) (Figure 3A). Their ADME information and 283 direct targets were summarized in Supplementary Tables 1 and 2, respectively. The meta-analysis of target overlaps for each herb, as well as the functional interaction among targets, were shown in Figure 3D. The same targets shared among herbs were linked by purple lines. Different targets in the same GO/KEGG items were linked by blue lines. There were little direct overlap, whereas much functional overlap among herbs. These might be due to different parts of the same biological processes regulated by different herbs. The enriched DisGenNET items in Supplementary Table 3 were then analyzed for depression related diseases (Figure 3B). Targets of FWP and each herb were mainly enriched in mood disorders, depressive disorder, and mental depression. The enriched diseases were more significant for total targets of FWP. These might be due to the synergistic effect of herbs in FWP. The enriched GO and KEGG items of total FWP targets were summarized in Figure 3C and Supplementary Table 4. The enriched cellular components (CC) included dendrite, synapse, and synaptic membrane. Molecular functions (MF) involved activity and binding of G protein-coupled receptor (GPCR), neurotransmitter receptors, and nuclear receptor. Moreover, biological processes (BP) results indicated that FWP might regulate depression-related signaling, such as GPCR, synaptic and various neurotransmitter signaling. The top enriched KEGG items were related to several critical depression-associated pathways, such as neuroactive ligand-receptor interaction, calcium signaling and synapse. These findings demonstrated FWP regulated synapse function, and neurotransmitter receptors like glutamate receptor to exhibit anti-depressive effects. The complex relationships between compounds in FWP and glutamate-related signaling were analyzed using a Compound-Target-Pathway Network (Figure 3E). There were 51 nodes (9 compounds, 38 targets, and 4 pathways) in this network connected by 103 interaction. Compounds in FWP regulated glutamate receptor signaling, glutamatergic synaptic transmission, glutamatergic synapse, and glutamate secretion. Saikosaponin A (B3) regulated glutamate receptor signaling. Hydroxycoumarin (AM1) regulated glutamatergic synaptic transmission and glutamate receptor signaling. 6-Gingerol (Z1) regulated glutamatergic synaptic transmission and glutamate secretion. Compounds from herb g and m could regulate all 4 related signaling.

### Evaluation of rat model of depression with LDSDS

**Figure 4:**
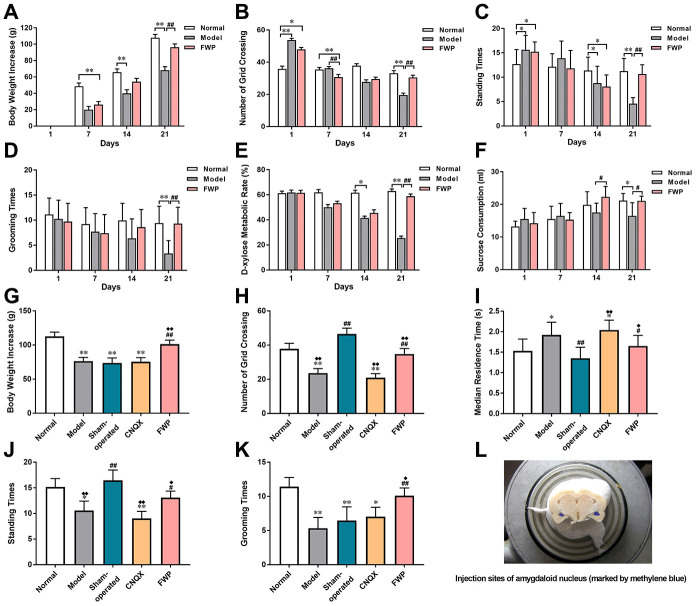
**Evaluation of chronic restraint stress induced rat model representing depression with liver-depression and spleen-deficiency syndrome, as well as effects of FWP on behavior changes.** For model evaluation experiment: (**A**) Body weight increase (g). (**B**–**D**) Number of grid crossing, standing times, and grooming times in open field test. (**E**) Urinary excretion rate of xylose test (%). (**F**) Sucrose consumption (mL). For FWP treatment experiments: (**G**) Body weight increase (g). (**H**–**K**) Number of grid crossing, median residence time (s), grooming times, and standing times in open field test. (**L**) CNQX injection sites in amygdaloid nucleus (marked by methylene blue). n=19 or 20 for (**A**–**F**), and n=13-15 for G-K. *P<0.05, **P<0.01 compared with the normal group; ^#^*P*<0.05, ^##^*P*<0.01compared with the model group; ^**♦**^*P*<0.05, ^**♦♦**^*P*<0.01 compared with the sham-operated group.

Normal rats kept a stable mental and physical state on day 1, 7, 14 and 21. During 21-day procedure, model rats showed sleep latency, reduced sexual/aggressive behaviours and self-care, impairments in place preference conditioning and brain stimulation reward, and frequent loose stool. The above changes were reversed on FWP-treated rats. Model rats exhibited significantly lower body weight increase compared with normal controls. FWP treatment could significantly reversed this lower pattern on day 21 (*P*<0.01, Figure 4A). When exposed to the OFT, model rats showed significant decreases in grid crossing counts, standing times and grooming times on day 21, which were alleviated after FWP treatment (*P*<0.01, Figures 4B–D). On day 21, compared with normal group, urinary excretion rate of xylose in model group decreased significantly, which were inhibited in FWP group (*P*<0.01, Figure 4E). Moreover, decreased sucrose preference is a anhedonia symptom representing clinical depression feature. Compared with normal rats, there was a significant decreased sucrose consumption in model rats, which was inhibited by FWP treatment (*P*<0.05, Figure 4F). Taken together, changes in model rats paralleled characteristic depression with LDSDS (i.e., depressed mood, psychomotor activity changes, anorexia, uncomfortable loose bowels).

### Effects of FWP on depressive behaviors in rats

The general state, body weight increase and behaviour in OFT were evaluated on day 21. Compared with model rats, there was not difference in sham-operated and CNQX groups, but significant alleviation in FWP group (*P*<0.01). On day 25, compared with normal rats, model rats exhibited depression-like behaviours with body weight increasing slowly (*P*<0.01, Figure 4G–4K). Compared with model, sham-operated rats suffered acute stress of operation and were irritable and hyperactive, with increased grid crossing counts, standing times and grooming times, as well as decreased residence times (*P*<0.01). These behavior changes were reversed significantly after FWP and CNQX treatment (*P*<0.05). In detail, FWP showed mild callback effect, whereas CNQX had an excessive effect. Moreover, compared with model, operation or CNQX treatment showed similar slow weight gain, while FWP treatment showed rapid weight gain on day 25 (Figure 4G). Therefore, FWP seemed to have a better regulatory effect.

### Effects of FWP on distribution of c-Fos, GluR1 and GluR2/3 immunoreactivity

**Figure 5:**
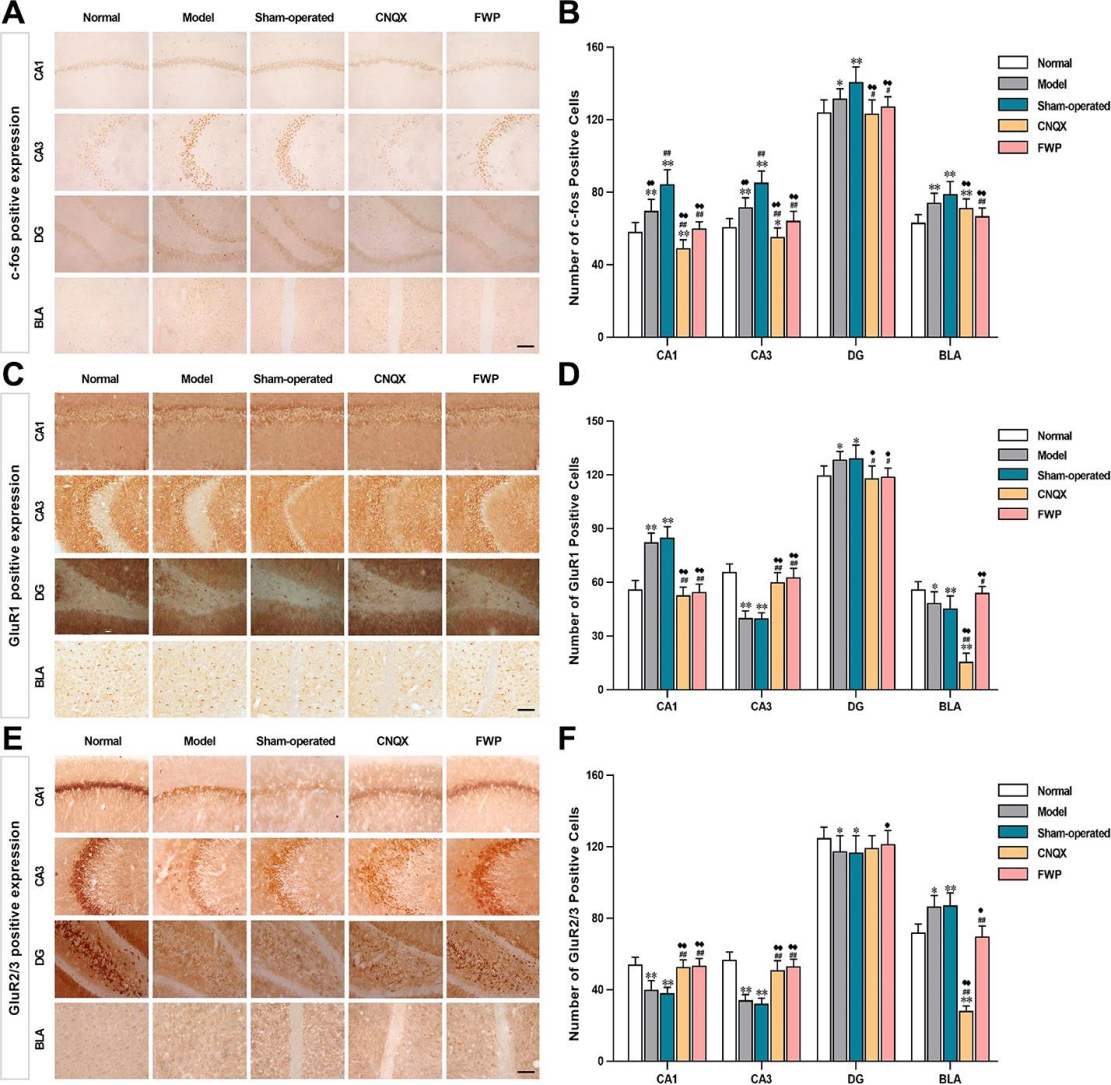
**Effects of FWP on c-Fos, GluR1 and GluR2/3 expression in hippocampus CA1, CA3 and DG, and amygdala BLA, in depressive rats with liver-depression and spleen-deficiency syndrome (×400, scale bar = 200 μm).** (**A**, **B**) The positive expression and positive cell of c-Fos; (**C**, **D**) The positive expression and positive cell of GluR1; (**E**, **F**) The positive expression and positive cell of GluR2/3. Results are presented as mean ± SD from 25 sections. *P<0.05, **P<0.01 compared with the normal group; ^#^*P*<0.05, ^##^*P*<0.01compared with the model group; ^**♦**^*P*<0.05, ^**♦♦**^*P*<0.01 compared with the sham-operated group.

The immediate early gene c-Fos is critical for neuronal plasticity. Neurons with c-Fos immunoreactivity were seen in hippocampus, bed nucleus of stria terminalis, amygdala, cingulate gyrus, marginal area and cortex. The stained neurons were round or oval-shaped, with the reaction product concentrated in nuclei. The membrane and cytoplasm were never stained (Figure 5A). Compared with control, there were significantly more neurons with c-Fos immunoreactivity in hippocampus (CA1, CA3 and DG) and amygdala (BLA) in model and sham-operated groups (*P*<0.05 or *P*<0.01), and slightly increased neurons with c-Fos immunoreactivity in FWP group (*P*>0.05). Compared with model and sham-operated groups, there were significantly less neurons with c-Fos immunoreactivity in CNQX and FWP group (*P*<0.05 or *P*<0.01, Figure 5B).

**Figure 6:**
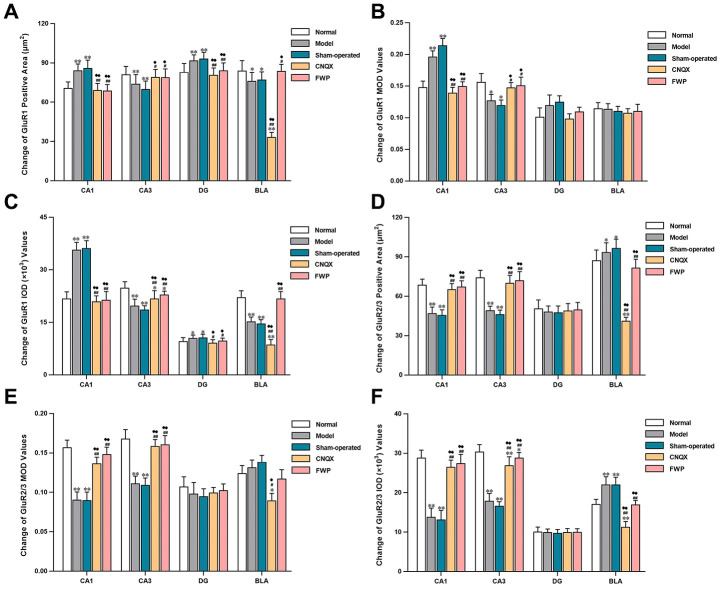
**Effects of FWP on AMPA receptor homeostasis in hippocampus and amygdala, in chronic restraint stress induced rat model representing depression with liver-depression and spleen-deficiency syndrome (×400).** (**A**–**C**) Change of GluR1 positive area (μm^2^), MOD, and IOD (×10^3^) values. (**D**–**F**) Change of GluR2/3 positive area (μm^2^), MOD, and IOD (×10^3^) values. Results are presented as mean ± SD from 25 sections. *P<0.05, **P<0.01 compared with the normal group; ^#^*P*<0.05, ^##^*P*<0.01compared with the model group; ^♦^*P*<0.05, ^♦♦^*P*<0.01 compared with the sham-operated group.

Specific GluR1 staining (brownish yellow) was concentrated in hippocampus and amygdala, while almost colourless in other parts (Figure 5C). The stained neurons were round, with orderly and closely arrangement. The membrane and fiber were darkly stained, and the cytoplasm and nuclei were not stained. Within the hippocampus, staining was especially prominent in the first and radiation layers of CA1, slight in CA3, and almost colourless in the transparent layer. The amygdala was more staining than cortex. Compared with control, there were significantly more neurons with GluR1 immunoreactivity in CA1 and DG, while significantly less in CA3 and BLA in model and sham-operated groups (*P*<0.05 or *P*<0.01). Compared with model and sham-operated groups, there were significantly less neurons with GluR1 immunoreactivity in CA1 and DG, while significantly more in CA3 and BLA in CNQX and FWP group (*P*<0.05 or *P*<0.01, Figure 5D). The results were similar in positive area change and integrated optical density (IOD) (Figure 6A, C), while a slightly different in mean optical density (MOD) (Figure 6B).

Specific GluR2/3 staining (brownish yellow) was concentrated in cerebral cortex, hippocampus, amygdala and hypothalamus, while almost colourless in other parts (Figure 5E). The stained neurons were circular, with darkly stained surface of nerve fiber and cell body. The cytoplasm and nuclei were not stained. Within the hippocampus, staining was especially prominent in soma layer, concentrated in the granular layer of DG, obvious in the initial/radiation/molecular layers, and almost colourless in the transparent layer of CA3. The amygdala was slightly more staining than cortex. Compared with control, there were significantly less neurons with GluR2/3 immunoreactivity in CA1, CA3 and DG, and significantly more in BLA in model and sham-operated groups (*P*<0.05 or *P*<0.01). There were slightly decreased neurons with GluR2/3 immunoreactivity in FWP group (*P*>0.05). Compared with model and sham-operated groups, there were significantly more neurons with GluR2/3 immunoreactivity in CA1, CA3 and DG, while significantly less in BLA in CNQX and FWP group (*P*<0.05 or *P*<0.01, except for result in DG of CNQX group: *P*>0.05, Figure 5F). For positive area change, IOD, and MOD, there were similar results in CA1, CA3 and BLA region, while no expression difference in DG region (Figure 6).

The similar immunohistochemical changes between FWP and CNQX groups suggested that FWP had a positive and balanced effect via antagonizing the AMPA receptor in amygdala. FWP inhibited AMPA receptor excitability, reduced the intensity of hippocampal stimulation and related damage.

### Effects of FWP on protein expression and phosphorylation of GluR1, GluR2 and GluR3

**Figure 7:**
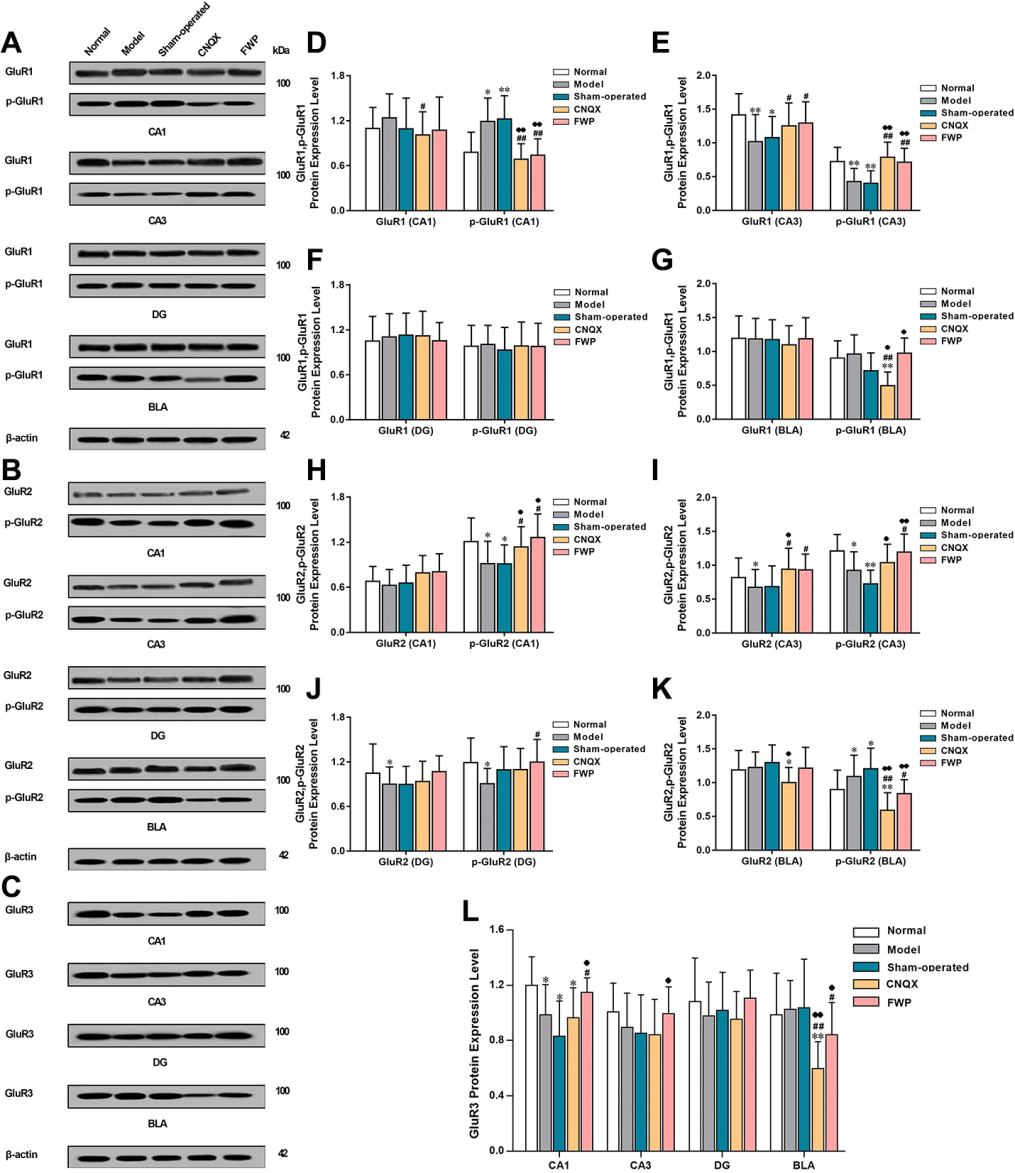
**Effects of FWP on protein expression and phosphorylation of GluR1, GluR2 and GluR3 in hippocampus and amygdala, in chronic restraint stress induced rat model representing depression with liver-depression and spleen-deficiency syndrome.** (**A**, **D**–**G**) protein expression and phosphorylation of GluR1. (**B**, **H**–**K**) protein expression and phosphorylation of GluR2 expression. (**C**, **L**) GluR3 expression (n=8). *P<0.05, **P<0.01 compared with the normal group; ^#^*P*<0.05, ^##^*P*<0.01compared with the model group; ^**♦**^*P*<0.05, ^**♦♦**^*P*<0.01 compared with the sham-operated group.

Compared with normal group, CRS in model group decreased GluR1 and p-GluR1 protein expression in CA3, GluR2 in CA3 and DG, p-GluR2 in CA1, CA3, and DG, and GluR3 in CA1 (*P*<0.05 or *P*<0.01), which were reversed by FWP treatment (*P*<0.05 or *P*<0.01, except for GluR2 in DG). Meanwhile, CRS increased p-GluR1 in CA1, p-GluR2 in BLA, which were also reversed by FWP treatment (*P*<0.05 or *P*<0.01). Compared with normal rats, rats in sham-operated group showed decreased expression of GluR1 and p-GluR1 in CA3, p-GluR2 in CA1 and CA3, and GluR3 in CA1 (*P*<0.05 or *P*<0.01), which were reversed by FWP treatment (*P*<0.05 or *P*<0.01, except for GluR2 in DG). Meanwhile, rats in sham-operated group showed increased expression p-GluR1 in CA1, p-GluR2 in BLA, which were also reversed by FWP treatment (*P*<0.05 or *P*<0.01, Figure 7). Results showed that FWP mainly regulated the phosphorylated form of protein GluR1 and GluR2.

### Effects of FWP on gene expression of GluR1, GluR2 and GluR3

**Figure 8:**
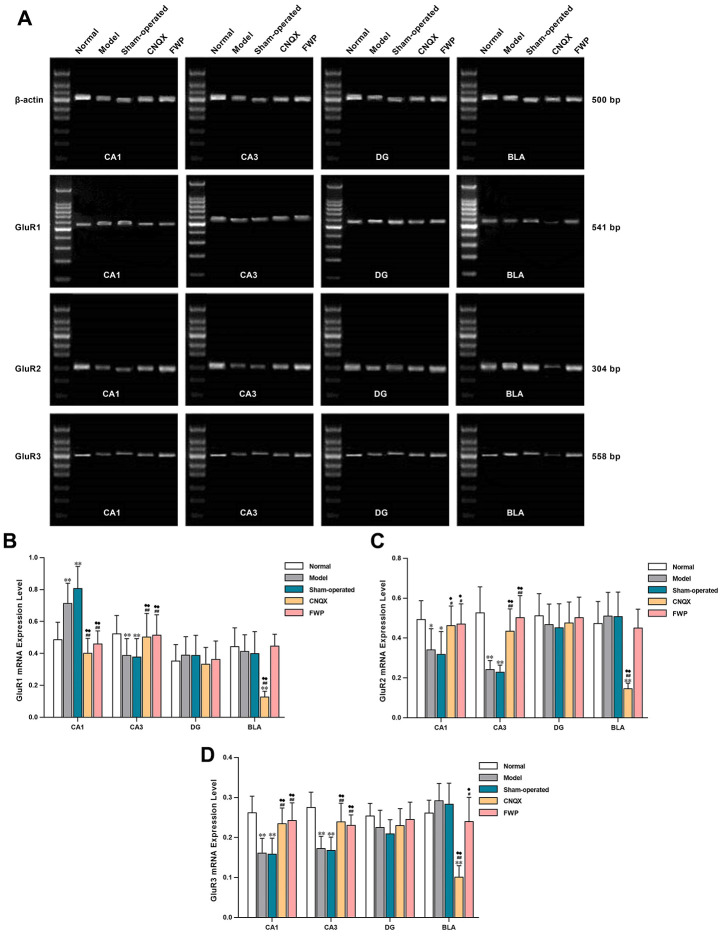
**Effects of FWP on gene expression of GluR1, GluR2 and GluR3 in hippocampus and amygdala, in chronic restraint stress induced rat model representing depression with liver-depression and spleen-deficiency syndrome.** (**A**) Electrophoresis of GluR1, GluR2 and GluR3. (**B**–**D**) relative quantitation of GluR1, GluR2, and GluR3 (n=5). *P<0.05, **P<0.01 compared with the normal group; ^#^*P*<0.05, ^##^*P*<0.01 compared with the model group; ^♦^*P*<0.05, ^♦♦^*P*<0.01 compared with the sham-operated group.

Compared with control, gene expression of the AMPA receptor subunits GluR1 were increased in CA1 and DG, and decreased in CA3 and BLA in model and sham-operated groups (*P*<0.01 in CA1 and CA3). The gene expressions of GluR2 and GluR3 were decreased in CA1, CA2, and CA3, and increased in BLA (*P*<0.01 in CA1 and CA3). The dysregulated expressions of GluR1, GluR2 and GluR3 were alleviated after FWP and CNQX treatment (*P*<0.05 or 0.01 in CA1 and CA3, Figure 8). Patterns of gene expression were consistent with protein expression and distribution for different AMPA receptor subunits.

## DISCUSSION

In the current study, we present evidence that FWP alleviates chronic stress induced depressive mood and physical changes, which mimics clinical LDSDS. The impaired synaptic plasticity and subsequent dysfunction of hippocampus (atrophy) and BLA (hypertrophy) are mainly improved by FWP treatment, which explains that limbic system is a major somatic organ affected in LDSDS. Interestingly, systematic bioinformatics analysis shows that different herbs in FWP synergistically regulate different parts of the same biological processes, which are mainly mapped into synapse function and neurotransmitter receptors. Nine compounds of 5 herbs in FWP regulates glutamate receptor signaling, glutamatergic synaptic transmission, glutamatergic synapse, and glutamate secretion. Our in vivo results are the first to demonstrate a potential mechanism of FWP regulating c-Fos expression via modifying the disturbed AMPAR homeostasis differently in different regions of hippocampus and BLA.

FWP is a classical TCM formula for depression with LDSDS, with many reports in experimental and clinical trials. Using optimized UHPLC-MS method, 23 compounds are firstly identified to clarify the complex bioactive ingredients of FWP. Previous studies have identified 8, 4, and 7 components in FWP, respectively [12, 19, 20]. Therefore, optimized extraction and LC methods here have supplied more information for potential constituents. Systematic bioinformatics is a powerful tool to integrate bioinformatics and experimental data for clarification of mechanisms of action of Chinese herbs [21]. Our results show that 283 targets of 17 compounds in FWP have mainly mapped into depression related disorders. These targets have functional overlap and regulate different parts of the same biological processes. This confirms a definite positive effect of FWP on depression in a multi-target/multi-component manner. CC enrichment results show that most targets regulated by FWP are located in dendrite, synapse, and synaptic membrane. Chronic stress is a major reason of depression, which cause disturbances in synaptic plasticity in hippocampus, such as long-term potentiation (LTP), along with behavioral defects including memory deficits [22]. The related damage to neuronal Nissl body, hypothalamic-pituitary-adrenal (HPA) axis, Ca^2+^ homeostasis, and neurotrophic factors (BDNF, NT3, TrkB) could be alleviated by FWP in previous studies [23, 24]. Our integrated results of BP, MF and KEGG enrichment also show that various neurotransmitter signaling, and related synaptic transmission are widely regulated by FWP. Glutamate is the primary excitatory neurotransmitter in neurons and glia. Enriched items here such as cAMP signaling, Ca^2+^ homeostasis, and cGMP-PKG signaling are related to glutamatergic neurotransmission [25–27]. Therefore, we construct a compound-target-pathway network to analyze potential effects of FWP on glutamate-related signaling. Compounds from herbs g, b, am, z and m have effects on glutamate secretion, glutamate receptor signaling, glutamatergic synaptic transmission, and glutamatergic synapse. Excitatory neurotransmission and its activity-dependent plasticity are largely determined by AMPARs [28]. Chronic stress-induced disruption of AMPARs includes it abnormal expression, trafficking, and calcium conductance at glutamatergic synapses, which contributes to synaptic plasticity at excitatory synapses [22]. Therefore, regulation of AMPARs is one potential mechanism for FWP against depression with LDSDS, which have not been systematically studied.

To fully discuss the relationship between expressions of AMPARs and depression with LDSDS, as well as possible effects of FWP on AMPARs, a rat model mimicking mood and physical changes of LDSDS is particularly important. One of our previous studies has compared Chinese formula targeting different depression subtypes in CRS induced rat model, and FWP has a comparative best effect [29]. Considering FWP is clinically effective for depression with LDSDS, CRS-induced rat model may bear a resemblance to the clinical picture of depression, and are evaluated further here. Here model rats show reduced sexual/aggressive behaviors and self-care (squealing, dark red eyes, light ear color, yellowish and dull hair, drooping beard, secretions in the corners of the eyes), loss of motor activity in OFT, impairments in place preference conditioning (stay in the corner quietly and motionlessly), poor appetite and digestion (weight loss, decrease sucrose consumption and xylose metabolic rate), and frequent loose stool. These physical and emotional changes are similar to most of clinical LDSDS symptoms, though pain is not evaluated. Clinical depression syndrome is heterogeneous with several combinations of changes in mood, sleep, energy, cognition, appetite and motor activity, which leads to a consensus view that the neurobiological basis for different depression subtype remains poorly understood [30].

C-Fos, an immediate early gene, is a tracing marker of neuronal activity [31]. Compared with normal rats, c-Fos expression increases in CA1, CA3, DG and BLA of CRS-induced rats. Compared with model rats, the operation-induced acute stress aggravates c-Fos expression in sham-operated rats. Consistent with this finding, the number of c-Fos positive cells depends on the intensity of stimulation [32]. Furthermore, AMPAR antagonist CNQX and FWP treatments decrease c-Fos expression. Thus, these findings indicate that AMPAR signaling regulates stress-induced c-Fos expression. FWP attenuate c-Fos expression by, at least partially, regulating AMPAR signaling. Increasing evidence has demonstrated that the subunit composition of AMPA receptors control receptor internalization and exocytosis, as well as glutamate excitotoxicity [33]. Of interest, significantly changed expression levels of AMPAR subunits have been detected in hippocampus and BLA in a region-specific manner.

The subunit composition determines synapse trafficking and functional properties of AMPAR [34]. Increased membrane expression of AMPARs mediates long-term potentiation (LTP) in hippocampus [35]. Homeostatic plasticity is also modulated via changing AMPAR trafficking processes in a subunit-specific and region-specific pattern [36]. GluR1 and GluR2 are common in the brain, and modulation of their membrane expression regulates AMPAR function. GluR1:GluR2 ratio determines Ca^2+^ influx or conductance of AMPARs. GluR1 mediates calcium ion influx, GluR2 renders the channel impermeable to calcium and diminishes cerebral excitotoxicity, both of which are important for LTP formation [37]. We find that chronic stress leads to increased GluR1 and reduced GluR2/3 protein expression in CA1 and DG, representing an increased GluR1:GluR2 ratio. However, a decreased GluR1:GluR2 ratio is seen in BLA. Here, we report that disturbed GluR1:GluR2 ratio contributes to CA1 and DG specific pyramidal neuronal loss and hippocampal atrophy, as well as BLA hypertrophy, which are reversed by FWP treatment. Our results are in line with previous conclusion, that altered GluA2 and GluA1 membrane expression would alter signal transduction which may contribute to cognitive and motor alterations [38]. Besides, the GluR3 subunit is known to have diverse neurophysiological impact, modulating oscillatory networks for sleep, breathing and seizure generation [39]. FWP here decreases the expression of GluR3 protein and gene in BLA region.

In addition, modulation of membrane expression of AMPAR by modulating phosphorylation of GluR1 and GluR2 subunits is an important mechanism in the modulation of glutamatergic neurotransmission and synaptic plasticity [40]. Phosphorylation of GluR1 at Ser845 by cAMP-dependent protein kinase (PKA) results in increased membrane expression and trafficking of GluR1. Here FWP reduces GluR1 phosphorylation in CA1. This result is in agreement with previous data showing that GluR1 phosphorylation occurs during synaptic potentiation [41], and is required for synaptic plasticity and spatial memory [42]. Phosphorylation of GluR2 at Ser880 by protein kinase C (PKC) results in decreased membrane expression, rapid internalization and trafficking of GluR2 [38, 34]. And FWP reduces GluR2 phosphorylation in BLA.

## CONCLUSIONS

**Figure 9:**
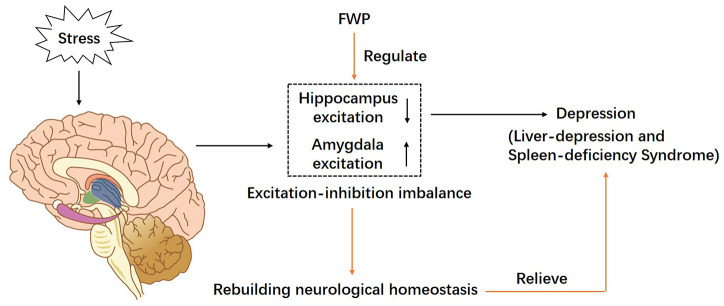
**A schematic diagram of proposed mechanism of FWP in chronic restraint stress induced rat model representing depression with liver-depression and spleen-deficiency syndrome.** FWP reconstructs neurological homeostasis via regulating the “excitation-inhibition” balance in amygdala and hippocampus.

In rats with liver-depression and spleen-deficiency syndrome, the function of amygdala (especially in the BLA region) was excited, while the function of hippocampus was inhibited. Antagonizing the AMPA receptor in the BLA region of amygdala, could alleviate the damage of the hippocampus. FWP, by antagonizing AMPA receptor in amygdala, can recover the “excitation-inhibition” balance in amygdala and hippocampus, and rebuild the AMPAR homeostasis, which may be an important pathway of FWP in the treatment of liver-depression and spleen-deficiency syndrome (Figure 9).

## MATERIALS AND METHODS

### Animals and reagents

Male SD rats (230±10 g, clean grade) were purchased from Vital River Laboratory Animal Technology Co. Ltd (SCXK (Beijing) 2002-0003, Beijing, China). Animals were housed in a quiet room on a 12-h light/dark cycle, maintaining the temperature (20–24°C) and relative humidity (30–40%). Standard laboratory chow and water were provided ad libitum. All animal experiments were approved by the Laboratory Animal Management and Ethics Committee of the Dongzhimen Hospital, and Beijing University of Chinese Medicine.

FWP consisted of eight herbs (Table 2), which were purchased from the Beijing Tongrentang Yinpian Co., Ltd. (Bozhou, China). The voucher specimens were identified by experts, and deposited at Beijing University of Chinese Medicine. The preparation method for FWP dry extract was shown in Supplementary Figure 1 with an extraction yield of 21.74%. FWP extract was dissolved in distilled water at a concentration of 0.3854 g/mL for intragastric administration. CNQX (Sigma, Milwaukee, USA) was dissolved in 20% DMSO/saline to a concentration of 1 mg/mL.

**Table 2:** Information of components in FWP.

**Chinese name**	**Botanical name**	**Common name**	**Weight (g)**	**Voucher Numbers**	**Part used**
Chai Hu	*Bupleurum chinense* DC. or *Bupleurum scorzonerifolium* Willd.	Radix Bupleuri	30	050304	Root
Dang Gui	*Angelica sinensis* (Oliv.) Diels.	Radix Angelicae Sinensis	30	050712	Root
Bai Shao	*Paeonia lactiflora* Pall.	Radix Paeoniae Alba	30	060109	Root
Bai Zhu	*Atractylodes macrocephala* Koidz.	Rhizoma Atractylodis Macrocephalae	30	050411	Rhizome
Fu Ling	*Poria cocos* (Schw.) Wolf	Poria	30	060515	Sclerotium
Zhi Gan Cao	*Glycyrrhiza uralensis* Fisch., *Glycyrrhiza inflata* Bat. or *Glycyrrhiza glabra* L.	Radix et Rhizoma Glycyrrhizae Praeparata cum Melle	15	060102	Root and Rhizome
Sheng Jiang	*Zingiber officinale* Rosc.	Rhizoma Zingiberis Recens	10	060307	Rhizome
Bo He	*Mentha arvensis* L.	Herba Menthae	10	060406	Aerial part

### UHPLC-MS

Five microliter of FWP solution, filtered through 0.22 μm membrane, was injected into an UltiMate 3000 LC system (Thermo Fisher Scientific, Bremen, Germany) on a C18 column (100 × 2.1 mm, 2.6 μm, Phenomenex Inc., Torrance, USA) at 35 °C. The mobile phase consisted of 0.1% formic acid (*v*/*v*) (A) and acetonitrile (B), and flow rate was 1 mL/min. The gradient used was: 0-30min 5%- 70%B, 30-35 min 70%-100% B, 35-39 min 100% B, 39-45 min 100%-5% B. The wavelength for detection was 254 nm. The mass spectrometer measurements were performed on a HR-ESI-MS detector with Thermo Q-Exactive system. The positive and negative electrospray voltages were 3.5 kV and 2.5 kV, and the capillary temperature was set at 300 °C.

### Systematic bioinformatics analysis for FWP

### Construction of FWP constituent and target database

Compounds identified by UHPLC-MS, were searched in NPASS [43] and PubChem Databases (https://pubchem.ncbi.nlm.nih.gov/). ADME properties were collected, comprising molecular weight (MW), lipophilicity descriptors (ALogP, MLogP, XLogP), hydrogen bond donor, hydrogen bond acceptor, polar surface area, rotatable bond, aromatic rings, heavy atoms and LipinskiFailure. Corresponding protein targets were retrieved, and then classified into each herb in FWP. Target source was mapped into Homo sapiens for further analysis.

### Interactions between targets

Protein–protein interaction data analysis was useful to characterize the molecular basis of disease. Interactions between targets were searched using Kyoto Encyclopedic of Genes and Genomes (KEGGs) [44], and Gene Ontology (GO) [45]. The correlation among targets of each herb was demonstrated by Circos diagrams using Metascape.

### Enrichment analysis for herb- disease correlation

Targets of FWP and each herb were mapped into DisGenNET database [46] through Metascape [47]. The accumulative hypergeometric p-values were calculated to filter the statistically enriched terms. Then, the enriched diseases related to depression were analyzed for each herb in FWP and visualized using heatmap of -Log P value.

### Functional enrichment analysis on GO and KEGG

Enrichment analysis was an effective method to increase the reliability of the identification of biological phenomena, resulting in meaningful annotation information. The “clusterProfiler” package in R was used to analyze enriched targets groups on KEGGs and GO items. *P* < 0.05 were considered statistically significant.

### Construction of compound-target-pathway network

To clarify the relationship between compounds in FWP and glutamate signaling, glutamate signaling-related genes were retrieved from the KEGG database. Intersections among compound-related genes and genes-related pathways were analyzed to create a Compound-Target-Pathway Network in CytoScape. The node correlation degree in the network was calculated using the STRING database [48]. The number of connections in network were calculated using Network Analyzer in Cytoscape and expressed as node size.

### Chronic restraint stress-induced rat model and treatment

### Model evaluation experiment

After 3 days of adaptive feeding, overactive or quiet rats were excluded. Rats were randomly divided into three groups (n=20): normal, model and FWP groups. All groups except normal received the chronic restraint stress procedure (8-11 a.m., 3h/day, 21 days, no access to food or water). The chest/ abdomen and four limbs were fixed by a soft bonds on a T-shaped bilayer restraint platform. Thirty minutes before restraint, rats received 3.854 g/kg•d FWP solution through gastric gavage in FWP group, or equal volume of saline in normal and model groups.

### FWP treatment experiment

Rats were randomly divided into 5 groups (n=15): normal, model, sham-operated, CNQX, and FWP groups. Rats received an above-mentioned restraint and treatment for 25 days. Rats in sham-operated and CNQX group received restraint and saline. On day 22, rats in CNQX group were injected with CNQX (0.5 μg in 0.5 μL) into bilateral amygdala, rats in sham-operated and FWP group were injected with 0.5 μL saline, while rats in normal and model group received no injection. During operation, 1 rat in sham-operated, 2 in CNQX, and 1 in FWP group died due to deep anaesthesia. For 300-350g SD male rats, the injection sites of amygdaloid nucleus (Figure 3L) were AP=-2.5, L=±4.4, DV =-7.9 [49]. Penicillin (160,000 units) were intra-peritoneally injected to prevent infection. On day 25, behaviour observation was performed. On day 26, rats were anaesthetized and transcardially perfused with heparinized saline (Supplementary Figure 2).

### Behavioral tests

### General observation

Before daily administration, rats’ mental state, posture, skin color, mobility, reactivity to restraint, eye fissure mucosa color, auricle color and feces were carefully observed.

### Weight gain

Rats was weighed on day 1, 7, 14, and 21. Weight gain was calculated with weight on day 1 as baseline.

### Open field test

On day 1, 7, 14 and 21, the open-field test was performed in a four-sided wooden enclosure, which was divided into 25 equal squares by black lines with grey floor and side walls (100cm×100cm×40 cm). Rat was gently placed in the central square. The number of grid crossing/median residence time, standing times, and grooming times were recorded by video tracking system for 5 min. The Observer 5.0 software (Noldus Information Technology B.V., Wageningen, Netherlands) was used to evaluate the autonomous activities, learning and memory, anxiety and other behaviour of rats.

### Sucrose consumption test

After 24h of water deprivation on day 1, 7, 14 and 21, rat was subjected to an individual metabolic cage in which 100 mL of 1% sucrose solution were placed. The consumption of 1% sucrose solution was calculated within 1 h.

### Urinary excretion rate of xylose test

On day 1, 7, 14 and 21, D-xylose-free urine was collected after 11h of food deprivation for each rat in an individual metabolic cage. Then rat was given 10% D-xylose solution (0.15g/100g). The urine was collected for the following 5h, and analysed using phloroglucinol method with absorbance measured at 554 nm [50]. Urinary D-xylose metabolic rate = D-xylose excreted from urine for 5 h (g)/amount of D-xylose administered (g) × 100%.

### Immunohistochemistry

The hippocampal CA1, CA3, DG and amygdala BLA regions were fixed in ice-cold 4% paraformaldehyde, cryoprotected with 30% sucrose solution, and sectioned into 30 μm slices. Sections were exposed to 3% H_2_O_2_, blocked with 10% normal goat serum, and incubated with c-Fos, GluR1, and GluR2/3 antibodies diluted in blocking sera (1:5000, 1:1000, and 1:1000). Then sections were incubated with biotinylated secondary antibodies (1:300, 1:200 and 1:200), stained using avidin-biotin-peroxidase complex (ABC, Sigma) and DAB solution, and dehydrated. Five coronal sections for each group were randomly chosen from equal levels of consecutive sections and viewed at 400× magnification (Nikon 4500 digital microscope). Image was processed as described in recent publications [51]. Positive cells were counted in a 200×100 μm^2^ region in CA1, and a 200×200 μm^2^ region in CA3, DG and BLA. Mean optical density (MOD), integral optical density (IOD) and area of positive substance (μm^2^) were quantified by the Image-Pro Plus 6.0 software.

### Western blot

The hippocampus and amygdala were dissected and CA1, CA3 and DG regions were separated, lysed by RIPA containing PMSF (Beyotime, Nanjing, China), homogenized and centrifuged. A total of 50 μg protein were resolved by 8% SDS-PAGE (Nanjing Jiancheng, Nanjing, China), transferred to a PVDF membrane, blocked with 5% milk, incubated with antibodies against pGluR1 (Ser845, Invitrogen, Carlsbad, USA), GluR1 (1:400, Invitrogen), pGluR2 (Ser880, Invitrogen), GluR2 (1:400, Invitrogen), GluR3 (1:400, Invitrogen), GluR2/GluR3 (Invitrogen), β-actin (1:5000, Cell Signaling Technology, Danvers, USA) at 4°C overnight, and hybridized with biotinylated-conjugated secondary antibody (1:5000, Vector Laboratories, Burlingame, USA) for 1 hours. The immunoreactive bands were visualized using an ECL system. The relative intensities of bands were quantified using Image J.

### RT-PCR

The hippocampal CA1, CA3, DG and amygdala BLA regions were collected, and RNA was extracted using TRIzol extraction Kit according to the instructions (Invitrogen). The cDNA was synthesized using cDNA reverse transcription kit (Fermentas, Burlington, Canada). Primer sequences were synthesized by SBS Genetech Co., Ltd. (Beijing, China) as follows: GluR1, Forward: 5'- GTCG TCCT CTTC CTGG TCAG CC -3', Reverse: 5'-GTGT CACA GGCT TTCG TTGC T-3', 541bp; GluR2, Forward: 5'-TTCC GTAA CCTT CGGA AGCA-3', Reverse: 5'-CAAG CCCA GACG TGTC ATTT C-3', 304bp; GluR3, Forward: 5'-TAGT CAGC AGAT TTAG CCCT TA-3', Reverse: 5'-TTTC CACC AACT TTCA TCGT AT-3', 558bp; β-actin, Forward: 5'-ATCA TGTT TGAG ACCT TCAA CA-3', Reverse: 5'-CATC TCTT GCTC GAAG TCCA-3', 500bp. PCR were performed following the instructions of AccessQuick RT-PCR system onestep kit (Promega, Madison, USA). Expression of target genes was corrected by the expression of β-actin and calculated using 2^-(Δ ΔCt)^ method. Finally, PCR product was electrophorized using 2.5% agarose gel and scanned by ImageMaster VDS system (Pharmacia Biotech, Inc., Uppsala, Sweden). The experiment was repeated three times.

### Statistical analysis

Data were expressed as mean ±SD, and analyzed using GraphPad Prism 7.0 (GraphPad Software Inc., La Jolla, United States). Normal distribution and homogeneity test between groups were performed. One-way analyses of variance (One-Way ANOVA) was used for comparison of multiple groups, followed by the least significant difference (LSD) post hoc test. The Kruskal-Wallis non-parametric H test was used when there was one inconsistency. *P*<0.05 was considered as statistically significant.

## Supplementary Material

Supplementary Figures

Supplementary Table 1

Supplementary Table 2

Supplementary Table 3

Supplementary Table 4
