# Supplementary material for: Free Wanderer Powder regulates AMPA receptor homeostasis in chronic restraint stress-induced rat model of depression with liver-depression and spleen-deficiency syndrome
Source: Aging (Albany NY). 2020 Oct 14;12(19):19563–84. doi: 10.18632/aging.103912 (PMC7732332; doi:10.18632/aging.103912)
Supplement: Supplementary Figures [file aging-12-103912-s001..pdf]

SUPPLEMENTARY FIGURES

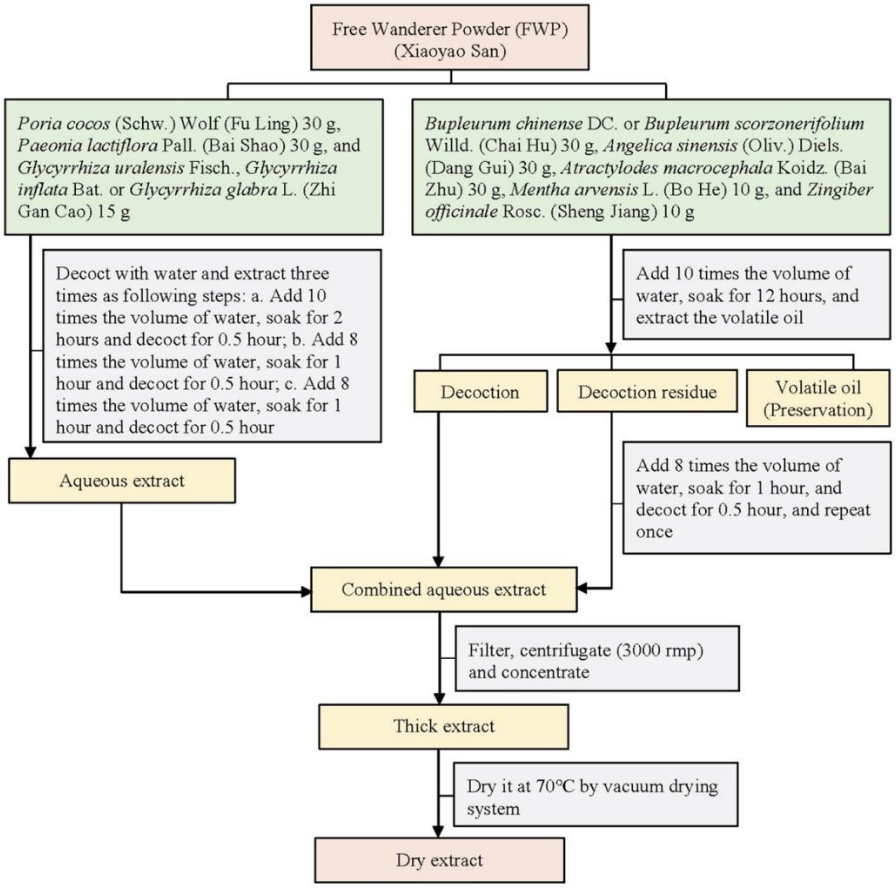

Supplementary Figure 1. Specific production technological process of Free Wanderer Powder (FWP) dry extract.

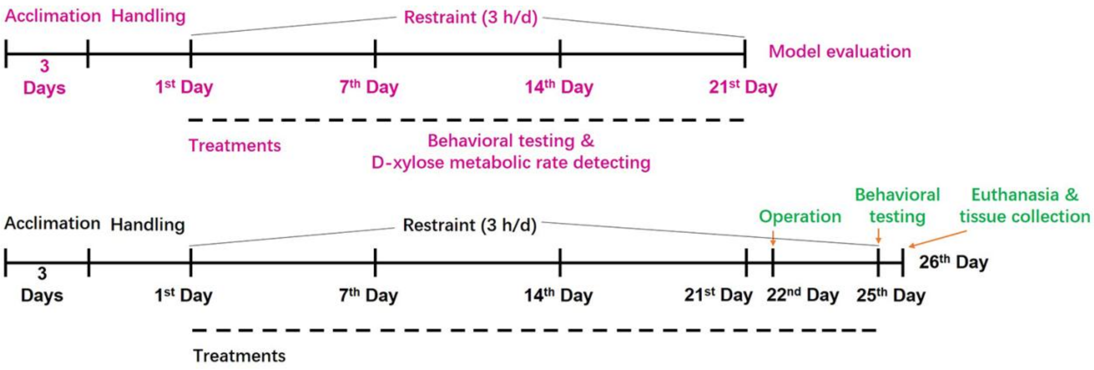

Supplementary Figure 2. Experimental design for model evaluation, efficacy and mechanism analysis. Rats were subjected to chronic restraint stress 3 h/day for 21 days and co-treated with saline, CNQX or FWP.
